# Orthodontists’ use of remote monitoring platforms pre-, amid, and post-COVID-19: a survey study

**DOI:** 10.1186/s12903-024-04245-2

**Published:** 2024-04-20

**Authors:** Sophie Logan, Christine A. Riedy, Kadriye Hargett, Negin Katebi

**Affiliations:** 1 Department of Developmental Biology, Harvard School of Dental Medicine, Boston, MA USA; 2 Department of Oral Health Policy and Epidemiology, Harvard School of Dental Medicine, Boston, MA USA; 3 Harvard School of Dental Medicine, Boston, MA USA; 4 Department of Developmental Biology, Harvard School of Dental Medicine, Boston, MA USA

**Keywords:** Orthodontics, COVID 19, Telehealth, Telecare

## Abstract

**Background:**

Did the COVID-19 pandemic affect orthodontists’ use of remote monitoring platforms? The goal of this research was to examine orthodontists’ experiences implementing remote monitoring platforms before, during, and after the initial COVID-19 lockdown.

**Methods:**

In this descriptive cross-sectional survey study, an electronic, anonymous questionnaire consisting of a series of 31 short-answer and multiple-choice questions was administered to an international sampling of practicing orthodontists. The target population in the study included currently practicing orthodontists who were graduates of an accredited orthodontic residency program. Participants were recruited in 2021 through collaboration with the American Association of Orthodontists (AAO) Partners in Research Program and the Harvard School of Dental Medicine Orthodontic Alumni Association. Descriptive analysis was conducted, reporting frequency (N and %) distributions for each question. The questionnaire aimed to describe whether orthodontists incorporated remote monitoring platforms into their practices, their experiences doing so, and if the COVID-19 pandemic influenced their use of these resources.

**Results:**

Orthodontists’ use of remote monitoring platforms was negligible prior to the pandemic; however, a quarter of surveyed orthodontists began using a remote monitoring platform during COVID-19 and nearly all respondents plan to continue using remote monitoring for the foreseeable future. Approximately half of orthodontists believe most patients’ treatment progress can be monitored to the standard of care between in-person orthodontic appointments using remote monitoring platforms. Half of the orthodontists who do not currently use a remote monitoring platform in their practice are interested in learning more about how to implement one.

**Conclusions:**

The COVID-19 pandemic led to an increase in the interest and adoption of remote monitoring platforms in orthodontic practices. Most orthodontists had not incorporated remote monitoring platforms into their practices prior to the COVID-19 pandemic. However, this study revealed that a subset of orthodontists utilized the pandemic as motivation to incorporate remote monitoring into their practices and an additional group of orthodontists were interested in incorporating one in the future. Remote monitoring platforms garnered interest and importance with the arrival of the COVID-19 pandemic and may only have an increasing role in the field in years to come.

**Supplementary Information:**

The online version contains supplementary material available at 10.1186/s12903-024-04245-2.

## Background

Over the last decade, many orthodontists have adopted advances in digital technology that allow an increase in office efficiency and improve the patient experience, including the use of clear aligners, three-dimensional (3D) printers, cone-beam computed tomography (CBCT), and intraoral scanners for digital 3D dental models [1–6]. The present study focused on one such advancement that has entered the market in recent years, remote monitoring platforms. Before these remote monitoring platforms were created, orthodontists could only evaluate the progression of their patient’s treatment via in-person appointments. Today, remote monitoring platforms can be used to the benefit of orthodontists and patients. Virtual check-ins can allow for fewer in-person appointments and an increased duration between in-office visits [7]. Further, the use of this technology can facilitate increased patient-provider communication, improved patient compliance, and less frequent emergency in-person appointments [8, 9]. Virtual monitoring can also allow patients to receive a more efficient, individualized, and satisfying care experience [8]. Notably, these platforms have proven capable of being incorporated into not only simple cases but also more complicated surgical orthodontic cases [7, 8, 10].

Remote monitoring platforms are defined in this study as any comprehensive Health Insurance Portability and Accountability Act (HIPAA)-secure digital platform that orthodontists can use to remotely monitor the treatment of their orthodontic patients. Further, these platforms enable patients to scan their teeth with a smartphone from the comfort of their homes and allow orthodontists to be able to monitor and communicate with patients virtually via a remote monitoring application. Dental Monitoring® and Grin® are two examples of remote monitoring companies that provide orthodontists with a means of virtually managing patient case progression. Dental Monitoring®, for example, converts the video scans patients take into 3D digital dental models for clinicians to evaluate [11, 12]. For the purposes of this study, the phrase “remote monitoring platforms” did not include Direct-to-consumer orthodontic companies or platforms that only allow patients to send photographs [13].

The need for having an alternative means of communication and evaluation of orthodontic treatment progression became evident during the COVID-19 pandemic, when social distancing guidelines and restrictions led to reduced office capacity [14]. Previous research outlines the potential benefits of incorporating orthodontic remote monitoring into practice but the willingness of orthodontists to incorporate this technology and the experiences of those who have done so already remains unknown. The aim of this study was to illuminate the past, current, and projected use of remote monitoring platforms in orthodontic practices, especially considering the influence of the COVID-19 pandemic. Through examining orthodontists’ usage of, experiences with, and opinions about remote monitoring platforms, such as Dental Monitoring®, the authors aimed to better understand the role that this key digital advancement will play in the field of orthodontics.

## Methods

In this descriptive cross-sectional survey study, an electronic, anonymous questionnaire consisting of a series of 31 short-answer and multiple-choice questions was administered to practicing orthodontists. The target population in the study included currently practicing orthodontists who were graduates of an accredited orthodontic residency program. Participants were recruited through collaboration with the American Association of Orthodontists (AAO) Partners in Research Program and the Harvard School of Dental Medicine Orthodontic Alumni Association. International American Association of Orthodontists (AAO) members were included in the study; however, AAO student members and retired or honorary AAO members were excluded to ensure the survey participants were most likely to be graduates of an accredited orthodontic residency and currently practicing orthodontics. The questionnaire aimed to understand whether orthodontists have incorporated remote monitoring platforms into their practices, their experiences doing so, and if the COVID-19 pandemic has influenced their use of these advancing resources.

After receiving approval from the Institutional Review Board (IRB) of the Harvard Faculty of Medicine (IRB21-0129) and the AAO legal/academic review teams, the recruitment email was sent out by the AAO to a subsection of their members (a random, deidentified sample and the maximum number they allow a single survey to be sent to) and the consent form was included in the survey as the first question. AAO has a membership of approximately 19,000 orthodontists; however, per the AAO Partners in Research program policy, the recruitment email could only be sent to approximately 2,300 members. Further, the AAO only allows surveys to be sent out once with one reminder email. The questionnaire was delivered via email to a random sample of 2,303 AAO listserv members on June 21, 2021, with a reminder email sent four weeks later. The AAO Partners in Research Program initially identified all members that met the inclusion criteria (i.e., active service, U.S. and international AAO members who were not students, retired, or honorary members), had not opted out of their emails and had not received a different Partners in Research survey within the last 45 days. Of that total, they then used the RAND function in Microsoft Excel to assign each eligible member a random number and selected 2,303 members to be sent the survey recruitment email and questionnaire. The Partners in Research Program did not factor member demographics into the sample selection.

Due to the low response rate, the questionnaire was sent out in a second wave in hopes of collecting more responses. A question was added at the beginning of the survey to confirm the participant had not taken the survey previously. If a participant indicated they had previously taken the survey, the survey would automatically end. The questionnaire was sent to a new random sample of 2,153 orthodontists from the AAO listserv on December 15, 2021, with a reminder email sent approximately 4 weeks later.

### Survey design

A panel of practicing orthodontists were recruited to conduct a cognitive interviewing technique to construct and validate the questionnaire. The content validation process included having the orthodontic experts independently complete the survey followed by one-on-one Zoom meetings between the PI and the experts, to conduct cognitive interviews to gather their feedback [15, 16]. The survey consisted of a series of 31 short-answer and multiple-choice questions about the orthodontists’ usage, experiences, and opinions of utilizing remote monitoring platforms to monitor clear aligner and braces treatment prior to, during, and following the COVID-19 pandemic. Examples of survey questions included multiple-choice questions such as, “In a practice where you work, [prior to/during] the COVID-19 pandemic, did you use a remote secure video conferencing platform (e.g., Zoom for Healthcare) for initial orthodontic patient consults or treatment planning appointments?” and “In a practice where you work, on average, what was the interval between clear aligner orthodontic appointments [prior to/during] the COVID-19 pandemic?”. Additionally, statements followed by Likert scale responses (ranging from “strongly agree (1) ” to “strongly disagree (5)”) were asked such as “Orthodontists can use remote monitoring platforms to monitor treatment progress between in-person orthodontic appointments to the standard of care for the majority of patients.” in which participants were asked to rate how much they agreed with the given statement. Moreover, participants were asked several demographic questions including their age, gender, and location of practice. The initial set of questions confirmed participants’ interest in completing the survey and inclusion criteria: currently practicing orthodontists who have graduated from accredited orthodontic residency programs. Following these eligibility questions, subsequent survey questions were optional.

Survey Administration: This study was designed as an anonymous, voluntary, electronic survey (Qualtrics, Provo, UT). An introductory email was sent to the AAO listserv by the AAO Partners in Research Program describing the study along with the link to the questionnaire. Interested participants clicked on the link and were directed to the Qualtrics survey, starting with the Longwood Medical Area (IRB21-0129) Exempt Human Research Consent statement. The survey was designed to take approximately 10 min to complete. No compensation was provided to participants.

Data Analysis: De-identified data were collected via Qualtrics and analyzed. Descriptive analysis was conducted and frequency (N and %) distributions for each question were reported.

## Results

Survey responses from both the first and second survey administration waves were combined. 180 responses were collected, and 146 responses were included. Those who did not consent, did not graduate from an accredited orthodontic residency, or did not currently practice orthodontics were excluded from the study. Additionally, 16 respondents did not answer any survey questions beyond the questions for inclusion. Table 1 describes the survey respondents’ demographics. Survey respondents were primarily male (68%, *n* = 88/130), had an average of 39 years in practice, and currently practice in 24 US states and territories and 32 countries. Some participants did not answer every question, therefore the number of responses (N) for specific questions varies.

**Table 1:** Survey respondent demographics *N* = 130

	Respondent Demographics
Demographics	
Gender	
Male Female Non-binary	67.7% (*N* = 88) 31.5% (*N* = 41) 0.8%<1% (*N* = 1)
Years Practicing Orthodontics	
Minimum Maximum Mean	2 years 58 years 39 years
Location of practice	
US Territories and States Represented (24)	California (8), Colorado (5), Missouri (5), Michigan (5), Georgia (4), Ohio (4), New York (3), Florida (3), Texas (3), South Carolina (3), Illinois (3), New Hampshire (2), Hawaii (2), Alabama (2), Massachusetts (2), Iowa (2), Peurto Rico (2), Pennsylvania (1), Arkansas (1), Nevada (1), South Carolina (1), Virginia (1), Maryland (1), Idaho (1), Kansas (1)
Countries Represented (32)	United States (66), Canada (12), Netherlands (5), India (4), Mexico (4), Greece (3), Italy (3), Spain (3), Pakistan (2), France (2), South Korea (2), United Kingdom (2), Australia (2), Brazil (2), Ireland (1), Haiti (1), Egypt (1), Nigeria (1), Switzerland (1), Columbia (1), Jordan (1), Malaysia (1), Czech Republic (1), Philippines (1), South Africa (1), Chile (1), United Arab Emirates (1), Armenia (1), Saudi Arabia (1), Israel (1), El Salvador (1), Kuwait (1)

**Fig. 1:**
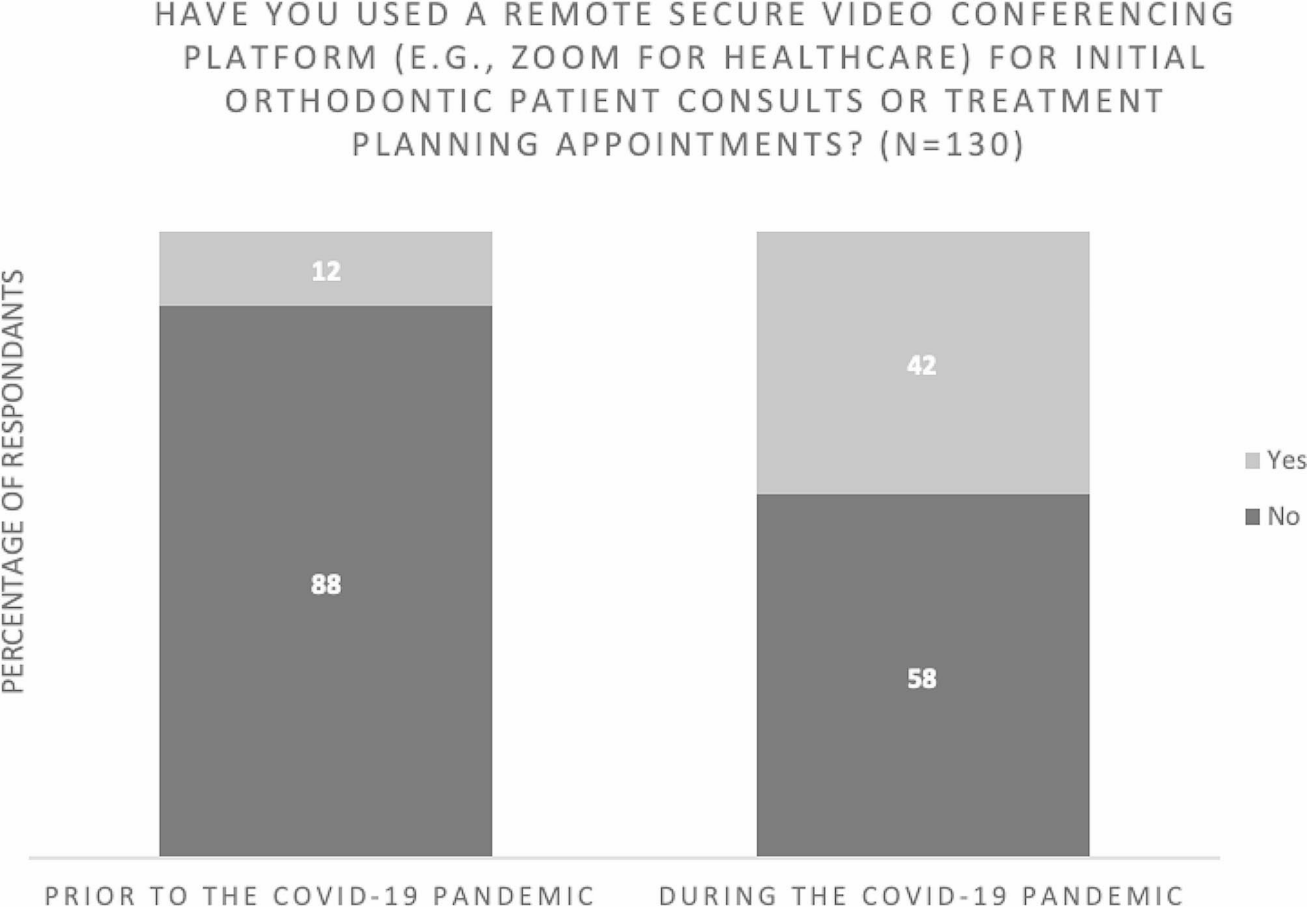
Influence of COVID-19 on use of remote secure video conferencing platforms (*N* = 130)

**Fig. 2:**
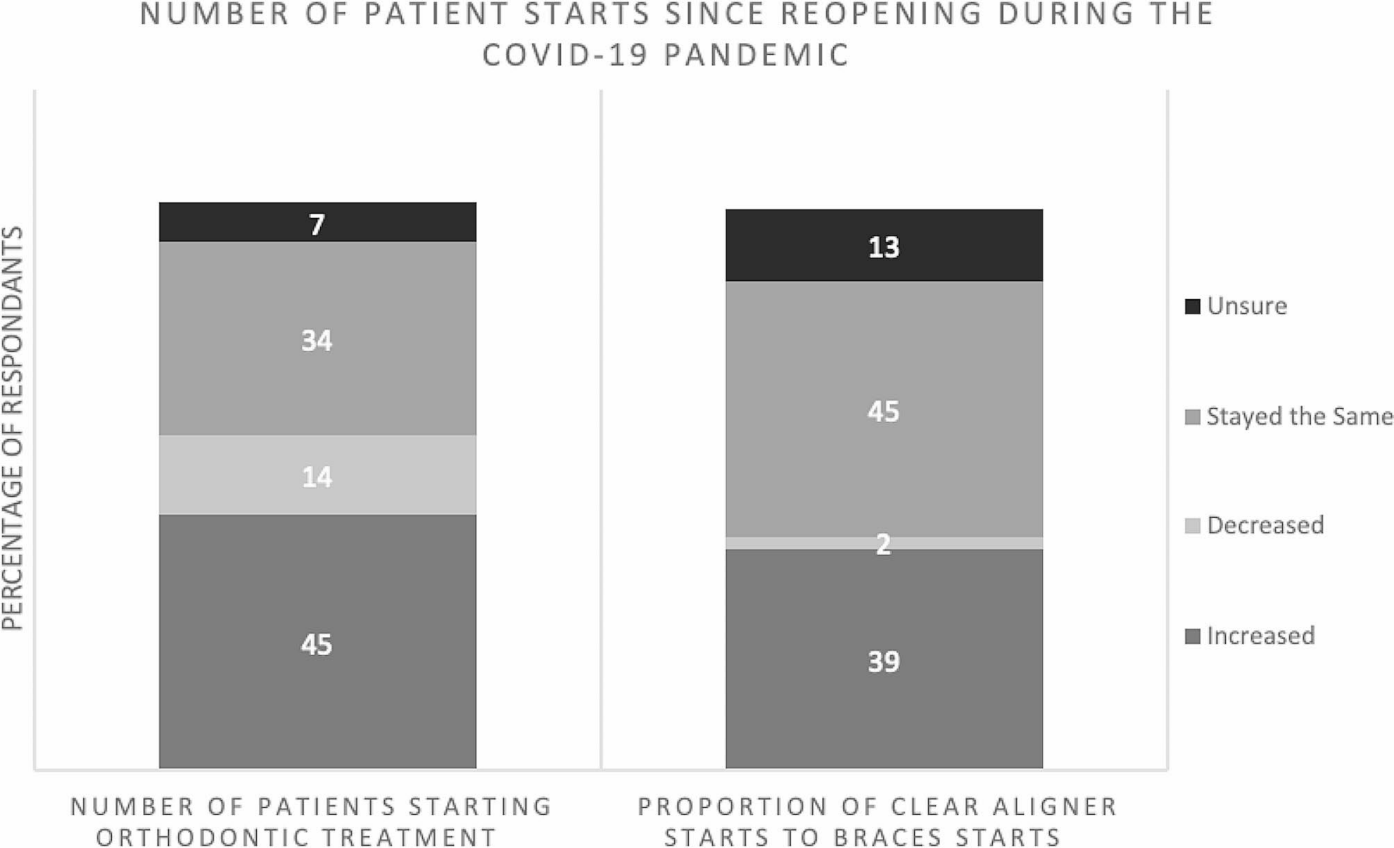
Influence of COVID-19 on patient case starts

Figure 1 demonstrates that very few respondents (12%, *n* = 16/130) used a remote secure video conferencing platform (e.g. Zoom for Healthcare®) for initial orthodontic patient consults or treatment planning appointments prior to the COVID-19 pandemic. However, during the COVID-19 pandemic, almost half (42%, 55/130) of the orthodontists reported using a remote platform. Since reopening during the COVID-19 pandemic, 45% (*n* = 58/130) of respondents noticed that the number of patients seeking orthodontic treatment increased and 39% (*n* = 51/130) noted that the proportion of patients starting clear aligner treatment relative to those starting braces treatment increased (see Fig. 2).

The most common interval between in-person appointments for both braces and clear aligner patients remained at 6–8 weeks both before the start of COVID-19 and since reopening (Table 2). The percentage of braces patients being seen every 6–8 weeks increased from 52% (*n* = 67/130) to 56% (*n* = 73/130) from before COVID-19 and since reopening. Prior to COVID-19, 41% (*n* = 54/130) of patients with fixed appliances were seen for in-person appointments every 3–5 weeks, the second most-common interval. That percentage decreased to 35% (*n* = 45/130) since reopening during the COVID-19 pandemic. Clear aligner patients were seen every 6–8 weeks according to 45% (*n* = 58/130) of the respondents prior to the COVID-19 pandemic, and since reopening during the COVID-19 pandemic, that number decreased to 43% (*n* = 56/130). Notably, the percentage of clear aligner patients being seen every 12 or more weeks for in-person appointments increased from 11% (*n* = 15/130) to 16% (*n* = 21/130) from before the COVID-19 pandemic and since reopening.

**Table 2:** Interval between clear aligner and braces orthodontic appointments prior to and during the COVID-19 pandemic

Interval between in-person appointments			
Prior to the COVID-19 pandemic:	% (*N*)	Since reopening during the COVID-19 pandemic:	% (*N*)
For clear aligner patients:		For clear aligner patients:	
3–5 weeks 6–8 weeks 9–11 weeks 12 + weeks	22% (28) 45% (58) 22% (29) 11% (15)	3–5 weeks 6–8 weeks 9–11 weeks 12 + weeks	15% (20) 43% (56) 25% (33) 16% (21)
For braces patients:		For braces patients:	
3–5 weeks 6–8 weeks 9–11 weeks 12 + weeks	41% (54) 52% (67) 7% (9) 0% (0)	3–5 weeks 6–8 weeks 9–11 weeks 12 + weeks	35% (45) 56% (73) 8% (11) < 1% (1)

The opinions of orthodontists regarding remote monitoring platforms are reported in Table 3. More than half of respondents 57% (*n* = 70/123) neither agree nor disagree that use of remote monitoring platforms increases patient compliance. The same number of respondents somewhat agree (38%, *n* = 47/123) and neither agree nor disagree (38%, *n* = 47/123) that the use of remote monitoring platforms decreases the overall number of in-person appointments. 29% (*n* = 36/123) of respondents somewhat agree that orthodontists can use remote monitoring platforms to monitor progress between in-person appointments to the standard of care and 18% (*n* = 22/123) strongly agree with that statement. Most respondents (20%, *n* = 24/123 strongly agree; 37%, *n* = 46/123 somewhat agree) believe patients appreciate the convenience of using remote monitoring platforms to track their care remotely, with only 8% (*n* = 10/123) somewhat disagreeing and 2% (*n* = 3/123) strongly disagreeing. A fifth of respondents (20%, *n* = 25/123) believe remote monitoring platforms can improve patient-provider communication in orthodontic settings with 37% (*n* = 45/123) somewhat agreeing with that statement.

**Table 3:** Respondents’ opinions of remote monitoring platforms (*N* = 123)

	Strongly Agree % (*N*)	Somewhat Agree % (*N*)	Neither Agree nor Disagree % (*N*)	Somewhat Disagree % (*N*)	Strongly Disagree % (*N*)
Using remote monitoring platforms					
Increases compliance in the majority of orthodontic patients. Decreases the overall number of appointments for the majority of orthodontic patients.	10.6% (13) 12% (5)	26.8% (33) 38% (47)	56.9% (70) 38% (47)	2.4% (3) 8% (10)	3.2% (4) 3% (4)
Orthodontists can use remote monitoring platforms to monitor treatment progress between in-person orthodontic appointments to the standard of care for the majority of patients.	18% (22)	29% (36)	28% (35)	16% (20)	8% (10)
The majority of orthodontic patients appreciate the convenience of using remote monitoring platforms to track their care remotely.	20% (24)	37% (46)	33% (40)	8% (10)	2% (3)
Remote monitoring platforms can improve patient-provider communication for the majority of orthodontic patients.	20% (25)	37% (45)	27% (33)	9% (11)	7% (9)

Of the 35% (*n* = 43/123) of respondents who currently use a remote monitoring platform in their office, 43% (*n* = 18/42) were using Dental Monitoring®, 2% (*n* = 1/42) were using Grin®, and 55% (*n* = 23/42) were using another platform. 83% (*n* = 25/30) of those respondents did not require that all patients use a remote treatment monitoring platform. Nearly all (94%, *n*=-32/34) respondents did not charge an additional fee for patients who use a remote monitoring platform. 69% (*n* = 29/42) of respondents who use a remote monitoring platform began using the platform between 2020 and 2021. It follows that 24% (*n* = 29/123) of respondents started using a remote monitoring platform between 2020 and 2021. 89% (*n* = 25/29) strongly or somewhat agreed with the statement that COVID-19 affected their decision to begin using a remote monitoring platform in their office, and 93% (*n* = 26/28) planned to continue using the platform after COVID-19. Respondents, when asked to select one or more reasons for using remote monitoring platforms reported that patients who are in clear aligner treatment, live far away, or have busy schedules are most likely to be encouraged to use the platform.

Out of the 65% (*n* = 80/123) of respondents who do not currently use a remote monitoring platform, half (50%, *n* = 40/80) reported that they are interested in learning more about using the platform for their patients and 39% (*n* = 31/80) would like to use remote monitoring platforms in the future. Survey respondents who used remote treatment monitoring platforms in the past (12.5%, *n* = 10/80) were asked to select one or more reasons they stopped using the platform. The most common reasons reported for discontinuing use were that the platform did not work as advertised (*n* = 4/19) and patients did not use the platform as instructed (*n* = 4/19). Other reasons reported were the high cost (*n* = 3/19), having insufficient staff to train patients (n-3/19), and having insufficient staff to monitor patients on the platform (*n* = 2/19). Three respondents indicated they discontinued use of the platform for another reason.

## Discussion

Remote monitoring platforms, such as Dental Monitoring®, Grin®, and Align Technology’s Virtual Care® have entered the market in recent years [14], allowing orthodontists to observe their patients between in-person office visits. During the COVID-19 pandemic, lockdowns became necessary in many parts of the world. Lockdowns necessitated decreases in live interactions, including dental appointments. In agreement with a survey by *Orthodontic Products*, which reported that only 29% of orthodontists had a remote monitoring platform in place prior to the COVID-19 pandemic [14], the present study shows that few orthodontists were using remote monitoring platforms prior to COVID-19. Further, we found that a quarter of all orthodontists surveyed began using a remote monitoring platform during the COVID-19 pandemic, with more than two-thirds of orthodontists who currently use remote monitoring platforms having started their use during COVID-19. This rise was likely in direct response to the urgent need for safe patient-provider communication during a period when in-person contact was deemed highly risky by public health officials. A dramatic increase of more than threefold was noted in the use of remote secure video conferencing platform (e.g. Zoom for Healthcare®) for initial orthodontic patient consults or treatment planning appointments during COVID-19 as compared to before the pandemic. Dhanasekaran et al. showed that 81% of orthodontists experienced disturbances in routine practice during the initial COVID-19 lockdown, with 65% of orthodontists communicating with patients via phone and video calls [17]. The present study found that prior to the pandemic, only 12% of orthodontists used a remote secure video conferencing platform (e.g., Zoom for Healthcare) for initial orthodontic patient consults or treatment planning appointments; however, 42% of orthodontists incorporated the platforms into their practice during the pandemic. The need for HIPAA-secure visual and verbal communication modalities was pressing, which explains the quick surge in use. Dental Monitoring® was the most popular remote monitoring platform among respondents, although a large proportion used another platform on the market. Around half of participants believe orthodontists can use remote monitoring platforms to observe progress between in-person appointments to the standard of care, patients appreciate the convenience of using remote monitoring platforms to remotely track care, and such platforms can improve patient-provider communication.

Additionally, a stark uptick was reported in the number of patients seeking orthodontic treatment after reopening during COVID-19 and an increase in the proportion of patients seeking clear aligner treatment compared to braces. Hansa et al. [8, 18] reported that Dental Monitoring® significantly reduced the number of in-person appointments. We found little difference in intervals between appointments prior to or after reopening from COVID-19, although there was a small rise in the number of orthodontists seeing clear aligner patients on 12 + week intervals. The increase in clear aligner patients might be attributed to the decrease of the burden of care with less frequent in-person appointments required by some practitioners.

Based on our findings, it is not common to require all patients in a practice to participate in remote monitoring. However, patients who live far away, are in clear aligner treatment, or have busy schedules may be most suited to using a remote monitoring platform. Despite the cost associated with remote monitoring platforms, respondents are not charging a fee for patients being monitored remotely. In contrast to the 57% of orthodontists surveyed by Severs et al. [19], nearly all orthodontists included in the present survey who started using a remote monitoring platform in 2020 or 2021 plan to continue using it going forward.

Study Limitations: One limitation of this study was that the AAO Partners in Research Program could not report duplicates between the recipient list of the June and December emails. Additionally, because the survey was sent to 210 (Institution Name Removed) Orthodontics alumni, some participants may have been surveyed twice. To mitigate potential bias, a question was added asking participants if they had previously taken the survey. An additional study limitation is the low-response rate of 3.86% (180/4666). While survey research can allow for the possibility of greater statistical power through a larger sample size, a disadvantage of an electronic format is the potential of non-response [20]. Remote monitoring is still a relatively new advancement in the field of orthodontics and some orthodontists who lack experience with remote monitoring may be generally unfamiliar with the technology. If the low response rate is due to under coverage bias in which only orthodontists familiar with remote mon1itoring responded to the questionnaire, the generalizability of our findings would be limited. The low response rate may have been improved had AAO allowed more than one reminder email to be sent to the study population. However, the total number of responses this study received is similar to that of other recent surveys of orthodontists [21–23]. Because this survey was voluntary, participants could exit freely or skip any question. Therefore, not all participants responded to every question that pertained to them, resulting in a variable number of responses. Another study limitation is that the survey is that orthodontists from over 30 countries responded to the survey. These orthodontists likely faced varying levels of restrictions associated with the pandemic. Participants each responded comparing their individual experiences prior to the pandemic, in the height of the pandemic, versus their experience post COVID-19. Participants who faced a complete lockdown may have been more motivated to incorporate remote monitoring into their practices than those in countries facing minimal pandemic-related restrictions.

New orthodontic technology will continue to emerge and further research will be required to determine what motivates orthodontists to incorporate new and advancing resources like virtual monitoring. However, amid and following the COVID-19 pandemic. remote monitoring platforms provided orthodontists with an additional means of patient-provider communication and can be utilized to provide a safe means of patient and provider communication during future periods of public emergency. Additionally, future work may further investigate whether certain orthodontic emergencies are best triaged by remote monitoring and which practice demographics are best associated with a need for remote monitoring implementation.

## Conclusions

Remote monitoring platforms garnered interest and importance with the arrival of the COVID-19 pandemic. The present study found that the COVID-19 pandemic has led to an increase in the interest in and adoption of remote monitoring platforms into orthodontic practices. Most orthodontists had not incorporated remote monitoring platforms into their practices prior to the shutdowns associated with the COVID-19 pandemic. However, this study revealed that a notable subset of orthodontists utilized the pandemic as motivation to incorporate remote monitoring into their practices and an additional group of orthodontists are interested in incorporating one in the future. Further, approximately half of orthodontists believe most patients’ treatment progress can be monitored to the standard of care between in-person orthodontic appointments using remote monitoring. While remote monitoring platforms garnered interest and importance with the arrival of the COVID-19 pandemic, they may only have an increasing role in the field in years to come.

### Electronic supplementary material

Below is the link to the electronic supplementary material.

Supplementary Material 1

## Data Availability

The datasets used and/or analysed during the current study available from the corresponding author on reasonable request.
