# Supplementary figures and images for: Orthodontists’ use of remote monitoring platforms pre-, amid, and post-COVID-19: a survey study
Source: BMC Oral Health. 2024 Apr 20;24:480. doi: 10.1186/s12903-024-04245-2 (PMC11032584; doi:10.1186/s12903-024-04245-2)

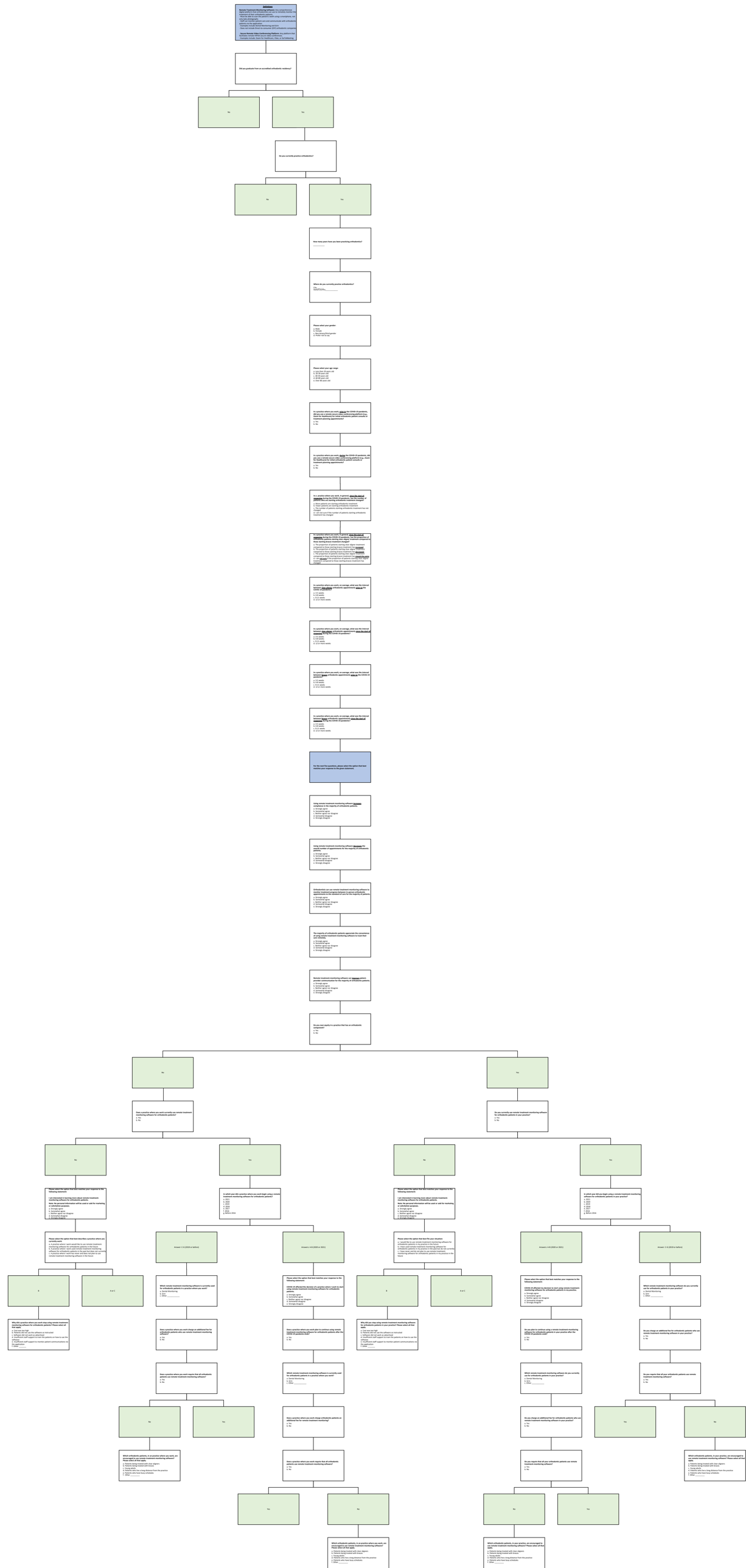

Supplement: Supplementary file 1 — Supplementary Material 1 [file 12903_2024_4245_MOESM1_ESM.pdf]
